# Leptomeningeal Metastases in Hormone Refractory Prostate Cancer

**DOI:** 10.7759/cureus.2470

**Published:** 2018-04-12

**Authors:** Jessica Y Ng, Justin Y Ng

**Affiliations:** 1 Surgery, Gold Coast University Hospital; 2 Medicine, Bond University

**Keywords:** metastases, leptomeninges, hormone-refractory prostate cancer, prostatic leptomeningeal metastases, prostate cancer

## Abstract

Leptomeningeal metastases from the prostate are extremely rare, with few cases described in the medical literature (antemortem prevalence of less than 0.03%). Prostatic leptomeningeal metastases harbour a poor prognosis with a median survival after diagnosis of 15.7 weeks. We present the case of an 82-year-old male with a history of hormone-refractory prostate cancer who presented to the emergency department with neurological symptoms.

## Introduction

In Australia, prostate cancer is the most commonly diagnosed cancer in men and accounts for 3,000 deaths per annum. The most common locations of metastases are bone, lung, and liver [1]. Leptomeningeal metastases are rare and usually are of gynaecologic origin. This is often due to ovarian cancer, with few reports of cervical or uterine cancer. It is important to recognise neurological signs and symptoms in patients with prostate cancer in advanced stages who have failed hormone therapy as their symptoms may be due to distant metastases.

## Case presentation

An 82-year-old male presented to the emergency department after a fall due to leg weakness. He reported a one-day history of associated gradual onset right occipital headache and left-sided arm weakness with concomitant paraesthesia, incoordination, and reduced grip strength.

On examination in the emergency room, the cranial nerves were grossly intact. The patient was able to demonstrate active movement against gravity and resistance in left upper and lower limbs. A left pronator drift was also present. Power in the right upper and lower limbs was unremarkable.

A differential diagnosis of subdural haematoma was considered at this time. The patient’s previous medical history included metastatic hormone-refractory prostate cancer, previously treated with a radical prostatectomy in 2005, followed by a bony metastatic recurrence in 2015 for which he received six cycles of docetaxel and goserelin. His prostate-specific antigen (PSA) was 0.06 ng/mL post-treatment. Despite this, the disease progressed with metastases to the ribs and lumbar spine. Subsequently, the patient received spinal radiation, abiraterone, and steroids for two months; enzalutamide was then given due to poor tolerability. Eventually, the enzalutamide was discontinued after four weeks in view of toxicity. He was commenced on metronomic oral cyclophosphamide, 50 mg daily, with prednisone, 5 mg twice a day. The patient went into remission and a subsequent PSA was 3.4.

**Figure 1:**
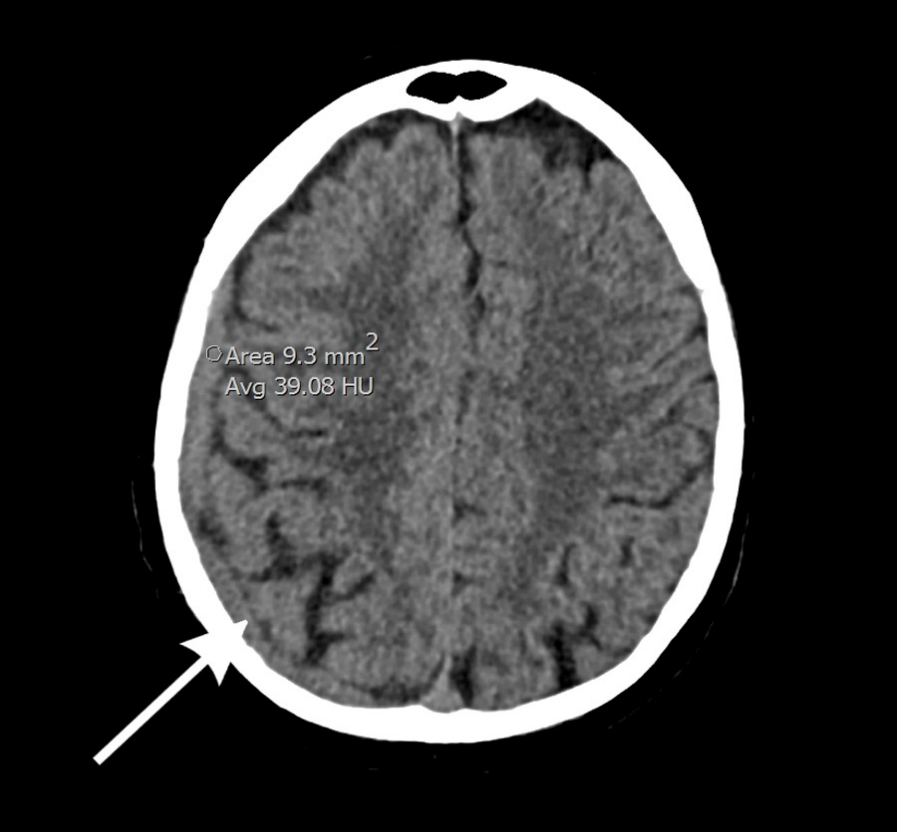
Computed tomography (CT) of the head shows a diffuse, enhancing dural thickening overlying the right hemisphere with focal areas of nodularity, suggestive of metastatic disease Associated mass effect is seen with 6 mm of left-sided midline shift.

**Figure 2:**
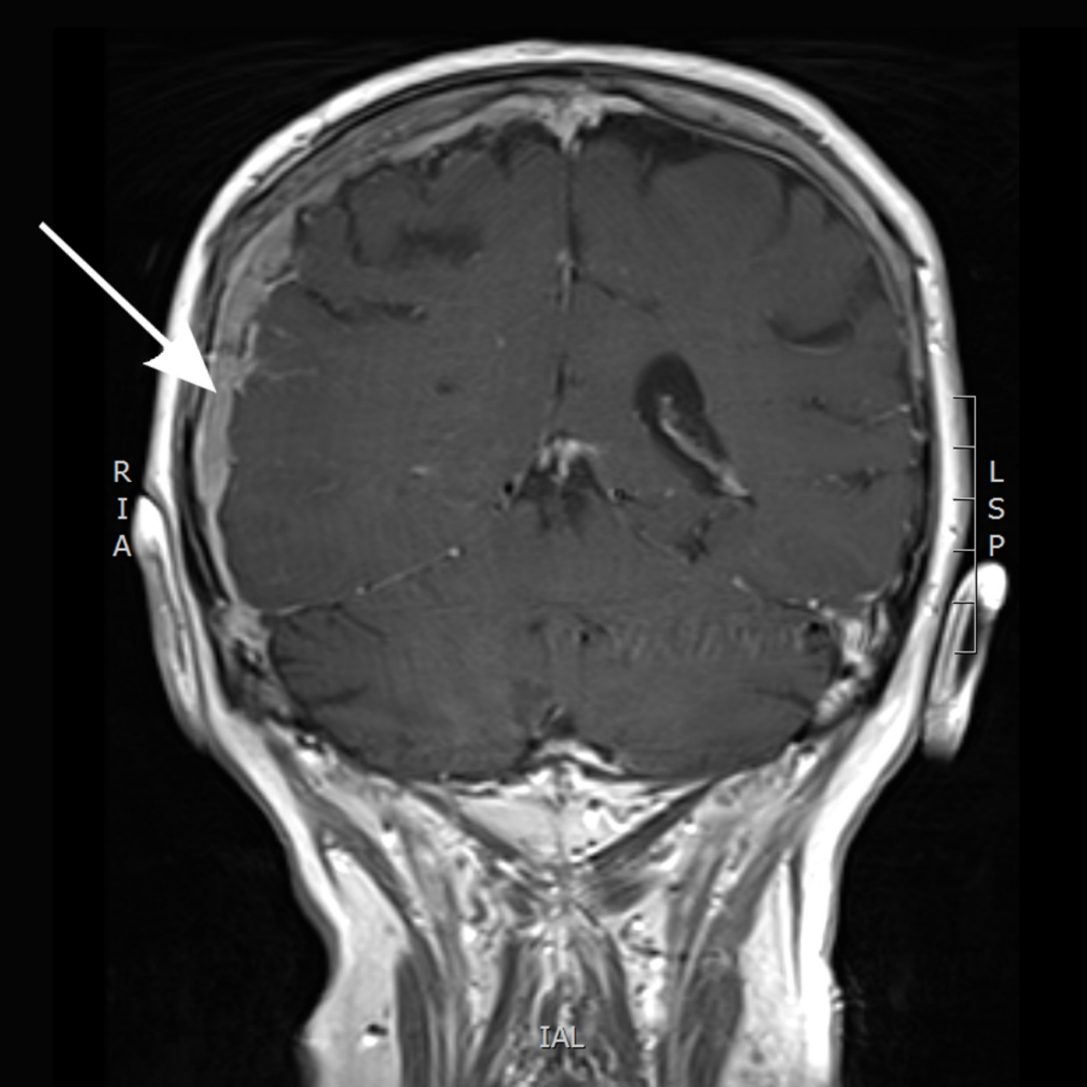
Magnetic resonance imaging (MRI) with gadolinium contrast showing pachymeningeal metastatic disease overlying the right cerebral convexity

Blood tests performed on his presentation to emergency revealed a PSA of 93. Full blood count and electrolytes were normal. Computed tomography (CT) of the head was performed which showed a diffuse enhancing dural thickening overlying the right hemisphere with focal areas of nodularity suggestive of metastatic disease. There was an associated mass effect with effacement of the right lateral ventricle and 6 mm of left-sided midline shift (Figure 1). This was believed to be unlikely due to a subdural hematoma, given the enhancement, and metastatic disease was thought to be more likely with his history. He then underwent magnetic resonance imaging (MRI) with gadolinium contrast which showed pachymeningeal metastatic disease overlying the right cerebral convexity, associated with metastatic marrow infiltration of the overlying calvarium and a small extracranial component. A leptomeningeal extension was also evident at the right frontoparietal junction (Figure 2).

Surgical management was not offered due to the extent of his disease. Palliative radiation therapy was recommended and he received 30 Gy in 10 fractions over two weeks to the whole brain, covering the calvarial disease and the meninges. Further palliative management was initiated with dexamethasone and hydromorphone. The patient began to rapidly decline with increasing falls and increasing analgesic requirement. His symptoms were managed with extensive involvement of a palliative team in a hospice setting. He passed away five months after diagnosis of leptomeningeal metastases.

## Discussion

Leptomeningeal metastases of prostate cancer are extremely rare; they are found in autopsy in approximately 5% of patients with systemic prostate cancer. In a study of genitourinary cancers with leptomeningeal metastases, Yu et al. reported an antemortem prevalence of less than 0.03% [2].

This disease harbours a poor prognosis with a median survival after diagnosis of 15.7 weeks [3]. Yu et al. suggested that the commonest route of involvement is from direct extension of skull metastases, followed by a haematogenous spread. Twenty percent of patients with leptomeningeal metastases are clinically asymptomatic [4].

Common symptoms previously described include that of increased intracranial pressure, resulting in headache, nausea and vomiting, and gait disturbances [3]. Diagnosis with neuroimaging in the form of MRI with gadolinium is preferred over the traditional method of cerebrospinal fluid (CSF) cytology due to its inferior morbidity for the patient and superior sensitivity (76–87%) [5]. In castration-resistant prostate cancer with metastatic skeletal disease, radium alpha 223 (a selective calcimimetic bone-seeking radionuclide for bone metastases) has shown a significant benefit with regards to pain reduction and improved quality of life and may be considered as a therapeutic option in patients who continue to progress after docetaxel and prednisone treatment [6]. Treatment for leptomeningeal disease is palliative with aims to improve quality of life and maintain neurological function, although external beam radiation, systemic, and intra-CSF chemotherapy (via surgical placement of an intraventricular catheter system) are possible [3].

Recent literature suggests that the incidence of brain and leptomeningeal involvement has increased since the use of docetaxel. Caffo et al. proposed that this is due to the inability of docetaxel to penetrate the blood-brain barrier, hence limiting its cerebral concentrations and thus rendering it less effective at eradicating tumour cells from the central nervous system (CNS) [6]. In the future, such cases may continue to rise secondary to the use of effective modern systemic treatments and prolonged patient survival or improvements in diagnostic techniques [3, 7-8].

## Conclusions

Leptomeningeal metastases from the prostate are extremely rare. It is important to recognise neurological signs in long-term patients who have failed hormone therapy as symptoms may be due to leptomeningeal metastases. Prompt recognition of metastases may allow for early palliative treatment and interventions to improve quality of life. There may be a trend towards increasing cases of leptomeningeal involvement in advanced stages of prostate cancer as survival can be prolonged by effective chemotherapeutic agents and diagnostic techniques have improved to detect disease.
